# Polychlorinated Biphenyls (PCBs) in the Environment: Occupational and Exposure Events, Effects on Human Health and Fertility

**DOI:** 10.3390/toxics10070365

**Published:** 2022-07-01

**Authors:** Luigi Montano, Concetta Pironti, Gabriella Pinto, Maria Ricciardi, Amalia Buono, Carlo Brogna, Marta Venier, Marina Piscopo, Angela Amoresano, Oriana Motta

**Affiliations:** 1 Andrology Unit and Service of Lifestyle Medicine in UroAndrology, Local Health Authority (ASL) Salerno, Coordination Unit of the Network for Environmental and Reproductive Health (Eco-FoodFertility Project), S. Francesco di Assisi Hospital, Oliveto Citra, 84020 Salerno, Italy; luigimontano@gmail.com; 2 PhD Program in Evolutionary Biology and Ecology, University of Rome Tor Vergata, 00133 Rome, Italy; 3 Department of Medicine Surgery and Dentistry “Scuola Medica Salernitana”, University of Salerno, Via S. Allende, 84081 Baronissi, Italy; cpironti@unisa.it (C.P.); mricciardi@unisa.it (M.R.); 4 Department of Chemical Sciences, University of Naples Federico II, Via Cinthia 26, 80126 Naples, Italy; gabriella.pinto@unina.it (G.P.); angela.amoresano@unina.it (A.A.); 5 INBB—Istituto Nazionale Biostrutture e Biosistemi, Consorzio Interuniversitario, 00136 Rome, Italy; 6 Research Laboratory Gentile, S.a.s., 80054 Gragnano, Italy; amabuono@libero.it; 7 Craniomed Laboratory Group Srl, Viale degli Astronauti 45, 83038 Montemiletto, Italy; dir.brogna@craniomed.it; 8 O’Neill School of Public and Environmental Affairs, Indiana University, Bloomington, IN 47405, USA; mvenier@indiana.edu; 9 Department of Biology, University of Naples Federico II, Via Cinthia 26, 80126 Naples, Italy; marina.piscopo@unina.it

**Keywords:** persistent organic pollutants, polychlorinated biphenyls, environment, occupational exposure, human exposure, health effects, fertility

## Abstract

In the last decade or so, polychlorinated biphenyls (PCBs) garnered renewed attention in the scientific community due to new evidence pointing at their continued presence in the environment and workplaces and the potential human risks related to their presence. PCBs move from the environment to humans through different routes; the dominant pathway is the ingestion of contaminated foods (fish, seafood and dairy products), followed by inhalation (both indoor and outdoor air), and, to a lesser extent, dust ingestion and dermal contact. Numerous studies reported the environmental and occupational exposure to these pollutants, deriving from building materials (flame-retardants, plasticizers, paints, caulking compounds, sealants, fluorescent light ballasts, etc.) and electrical equipment. The highest PCBs contaminations were detected in e-waste recycling sites, suggesting the need for the implementation of remediation strategies of such polluted areas to safeguard the health of workers and local populations. Furthermore, a significant correlation between PCB exposure and increased blood PCB concentrations was observed in people working in PCB-contaminated workplaces. Several epidemiological studies suggest that environmental and occupational exposure to high concentrations of PCBs is associated with different health outcomes, such as neuropsychological and neurobehavioral deficits, dementia, immune system dysfunctions, cardiovascular diseases and cancer. In addition, recent studies indicate that PCBs bioaccumulation can reduce fertility, with harmful effects on the reproductive system that can be passed to offspring. In the near future, further studies are needed to assess the real effects of PCBs exposure at low concentrations for prolonged exposure in workplaces and specific indoor environments.

## 1. Introduction

Chemicals detected in the environment, with the peculiarity of having long half-lives in soils (generally years), sediments, air (several days) or biota are defined as persistent organic pollutants (POPs). In recent years, studies on chemistry and effects of POPs are increasing and this topic became a fascinating scientific research area [1,2,3,4]. Several POPs are listed under the Stockholm Convention on Persistent Organic Pollutants, including chlorinated (and brominated) aromatics, such as polychlorinated biphenyls (PCBs), polychlorinated dibenzofurans, polychlorinated dibenzo-p-dioxins and polybrominated diphenyl ethers, as well as several organochlorine pesticides (e.g., dichloro-diphenyl-trichloroethane and its metabolites, chlordane, toxaphene, etc.) [5]. Moreover, these POPs can be easily absorbed by microplastics, which become vectors of these organic contaminants, facilitating their dispersion in different environmental compartments [6,7,8].

**Figure 1:**
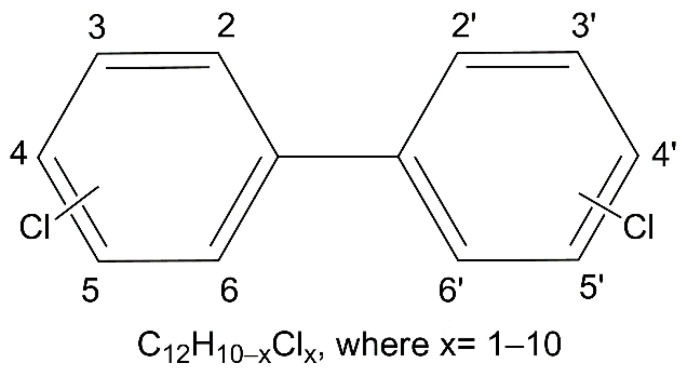
General structure and formula of PCBs. Chlorine atoms can replace hydrogens in different positions of the aromatic rings.

Polychlorinated biphenyls are persistent organic pollutants that have a negative impact on the ecosystem and all living beings and continue to represent a serious risk to human health [9]. Large-scale production of PCBs started in 1945. Thanks to their chemical characteristics and thermal stability, they were used as dielectric fluids (in transformers and electric capacitors) and as additives for pesticides, flame retardants, insulators, paints, glues and printing inks [10,11]. PCBs are obtained from oil and tar, from which benzene is extracted, and then transformed into biphenyl, which is subsequently chlorinated to polychlorinated biphenyl. The chemical structure is characterized by the presence of two aromatic rings on which there are 1 to 10 chlorine atoms (Figure 1).

The various combinations determined by the number and position of the chlorine atoms result in 209 different compounds called congeners. The IUPAC (International Union of Pure and Applied Chemistry) nomenclature assigns each congener a number between 1 and 209 [12]. The chemical and physical characteristics of PCBs differ considerably among congeners leading to a high structural variability, which has direct consequences on persistence and bioaccumulation. Based on the number of chlorine atoms, PCB congeners can be divided into low-chlorine PCBs if they contain four or fewer chlorine substituents, and high-chlorine PCBs if they have more than four chlorine atoms [13]. These differences determine their environmental availability and their routes of exposure. High-chlorine PCBs are relatively non-volatiles, mainly present in food and more persistent than low-chlorine PCBs, thanks to their resistance to metabolic degradation. Conversely, low-chlorine PCBs, often referred to as transient or episodic forms due to their relatively short half-lives, are semi-volatiles and are rapidly metabolized [14,15,16]. Low-chlorine PCBs are the main PCBs found in indoor [17] and outdoor [18,19,20] air, especially in large cities and urban industrial areas, e.g., in the air of major cities [16,21,22,23,24]. 

Among all 209 compounds classified as PCBs, 12 (i.e., PCB 77, 81, 105, 114, 118, 123, 126, 156, 157, 167, 169 and 189) have physio-chemical and toxicological properties comparable to those of dioxins and furans and are, therefore, called dioxin-like PCBs. Being all coplanar, they can bind to the aryl hydrocarbon receptor (AhR), which is the canonical receptor for 2,3,7,8-tetrachlorodibenzo-p-dioxin [25]. Importantly, non-dioxin-like PCBs account for a significantly high proportion of PCBs found in human serum, adipose tissue and breast milk [26]. Moreover, PCBs could be also divided into “legacy” and “non-legacy” or “contemporary” [27]. The latter are formed as inadvertent by-products during the production of currently sold paints, inks and dyes, so are released into the environment unintentionally [28,29,30,31]. 

Although their use was banned in the 1970s, their resistance to chemical and thermal degradation results in bioaccumulation in marine organisms and humans. Thus PCBs continue to be an environmental and human health concern [13,32]. The occurrence of several chronic diseases, such as endocrine dysfunction, type 2 diabetes, cardiovascular disease, obesity, liver disorders, and neurological deficits, have been associated with exposure to PCBs [33]. Moreover, they affect the immune, reproductive, nervous and endocrine systems and are carcinogenic [34,35,36].

Considering the growing interest in this research field, the present review aims to discuss the accumulation of the different PCBs based on their chemical-physical and toxicological characteristics, routes for human exposure (both environmental and occupational) and subsequent effects on human health.

## 2. Methodology

Based on the recent scientific literature related to PCBs occurrence in the environment, this review had three main objectives: (1) describe the different routes for human exposure to PCBs; (2) highlight the importance of PCBs level of contamination in workplaces for occupational exposure; (3) discuss the effects on human health due to exposure and bioaccumulation of PCBs. The keywords “persistent organic pollutants”, “polychlorinated biphenyls”, “environment”, “occupational exposure”, “human exposure”, “health effects” and “fertility” were selected individually or jointly to search for relevant information on the Web of Science, Scopus and Google Scholar. The literature search covered articles published between 1990 and 2022.

## 3. Human Exposure and Bioaccumulation of PCB

### 3.1. Routes for Human Exposure

**Figure 2:**
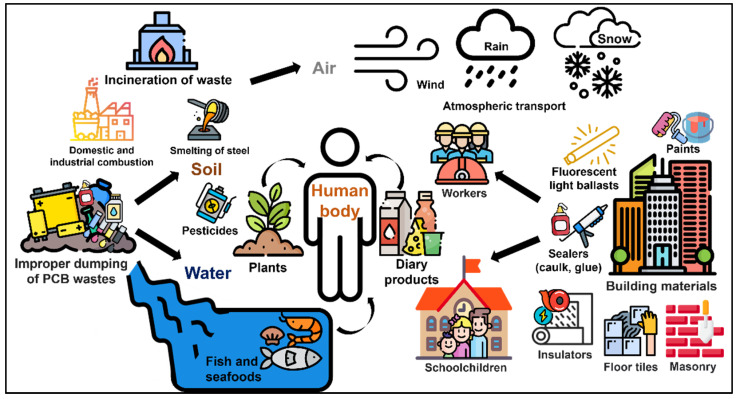
PCB sources and routes for human exposure.

PCBs can be generated from thermal processes, mainly waste incineration, but also steel smelting and domestic and industrial combustion of coal and wood (Figure 2). However, it must be considered that PCB congeners combined emissions generally contribute only a few percent to total air POPs emissions from domestic coal and wood combustion, which are mainly made of polycyclic aromatic hydrocarbons and particulate matter [37,38]. The main PCBs emission sources are electricity production (principally due to the consumption of coal as fuel), steel production and incineration, including that of waste. In addition to point sources, PCBs can be transported for long distances via deposition and resuspension processes (i.e., dry fallout and vapor deposition) that can be described as the grasshopper effect [39,40]. Human exposure to PCBs can derive from different sources, such as dietary intake, inhalation, ingestion of dust and dermal contact [41]. In Figure 2 are shown the main routes for the release of PCBs into the environment and relative human exposure routes.

The main exposure route to PCBs for humans is the consumption of contaminated food, mainly fish, seafood and dairy products [42,43]. In fact, numerous studies have shown that animal products containing fats are the most contaminated food sources, so their intake represents one of the principal routes of exposure to these POPs. Due to frequent health recommendations regarding fish consumption, determining the contribution to dietary intake of chemical contaminants, such as PCBs, is a matter of particular concern. Indeed, it was concluded that population groups that frequently consume large quantities of dietary items rich in fats could experience significantly higher health risks from exposure to PCBs and POPs in general [44,45,46]. Epidemiological studies have shown a correlation between the consumption of contaminated fish and the increase in the serum concentrations of PCBs [45]. Very recently, the association between dietary intake and PCBs serum levels was examined, revealing that body mass index can modify this association with a stronger connection among normal/underweight individuals [47].

Studies that investigated PCBs human intake through food consumption are mainly focused on fish, followed by meats (beef, pork, and poultry), dairy products and chicken eggs. Recently, it was found that salmon is the food item that contains the highest amount of POPs including PCBs, followed by canned tuna, beef steak, butter and fried chicken [48]. However, a reduction in PCB concentration in food was observed over the last 20 years, indicating a decrease in dietary exposure over time [48]. Furthermore, it has to be underlined that different human populations may differ in their exposure susceptibility to PCBs because of their differences in terms of lifestyle, living environment and dietary habits [49].

A second route for PCB exposure, albeit often overlooked, is inhalation, mainly indoors and to a lesser intent outdoors. This applies particularly to densely populated industrial areas and homes and buildings that have been constructed using PCBs in sealants and other building materials [24,50,51]. In fact, the highest concentrations of PCBs are found in the indoor and outdoor air of industrialized and densely populated urban areas in the cities of Chicago, Milwaukee, Toronto, Philadelphia and New York [23,52,53,54,55,56,57]. Since the volatilization of airborne PCBs is temperature dependent, this phenomenon can lead to their release from environmental or reservoirs, such as rivers, lakes, landfills or contaminated building materials [24,26,58]. Airborne PCBs also include most of the recently discovered non-legacy PCBs. Non-legacy PCBs are present in both indoor and outdoor environments and they can ultimately accumulate in the bodies of exposed populations. Many studies have reported the presence of non-legacy PCBs in air samples around the world. Volatilization from commonly used paints is the most likely source of these contaminants [28,59,60]. In 2010, over 50 non-legacy PCBs were detected in pigments employed in household paint [29]. Several studies have analyzed the effect of indoor air on PCB contamination. This is due to the fact that levels in indoor air may be several orders of magnitude greater compared to outdoor air and that people spend much more time indoors than outdoors. In order to get more information about potential health hazards due to indoor air PCBs in the literature, some studies investigated the PCB indoor concentration in schools, as well as the blood levels. In different schools the blood analyses indicated an increase in teachers from a school with heavy contamination of low chlorinated PCB [61,62,63,64]. 

Among all the exposure pathways, the dietary intake of PCBs continues to be the major one, despite having observed a recent increase in contributions from indoor air inhalation. The other possible exposure routes (dermal contact and ingestion of dust) do not contribute significantly to the overall PCB exposure [41]. If the decrease in PCB concentrations in food observed in the last years continues, PCB inhalation could become comparable to dietary exposure in the near future [48]. This trend is related to the fact that legacy high-chlorine PCBs can be metabolized and eliminated from the food chain, whereas non-legacy PCBs are still inadvertently produced in modern paints and consumer items [13,29,65]. 

### 3.2. Occupation and Exposure Events in Workplaces

The potential health risks posed by pollutants, such as PCBs in the indoor environment are of great concern [66,67,68,69,70]. People generally spend more than 90% of their time indoors, between their home and their workplace. For many years there has been a large use of PCBs in the production of materials and/or objects typically found in indoor environments, such as building, sealing and caulking and materials, fluorescent lighting fixtures, electrical equipment, plasticizers, surface coatings, paints and ink [71,72]. Therefore, they could still be released in the indoor environment, absorbed as indoor dust and bio accumulated by people via non-dietary ingestion and inhalation pathways. 

Indoor PCBs inhalation is a cause for concern in schools and other buildings (e.g., offices) constructed and refurbished especially from the 1950s to the late 1970s, as demonstrated by several studies investigating indoor PCB exposure in the United States and Europe [17,61,73,74,75,76,77,78,79]. During this period, caulking compounds (waterproofing technique), sealants and other building materials (e.g., fluorescent light ballasts) contained high levels of PCBs, and affected buildings still represent a major problem for chronic inhalation. The most relevant data concerning PCB air pollution in indoor environment are reported in Table 1.

High concentrations of low-chlorine PCB congeners were detected in the indoor air of polluted schools in Germany [61]. Furthermore, there was a significant correlation between PCB exposure and increased blood PCB concentrations in teachers who had worked in these contaminated school buildings. 

In a school in the town of Columbus Junction (Iowa) different PCB concentrations were found based on construction year, which in turn relates to different use of PCB-containing building materials during the time [95]. The highest concentrations were detected in the rooms in the oldest wing of the building (e.g., 39.2 ng m^−3^ in the math room PCBs constructed before 1920), while the lower values were measured in rooms built more recently (e.g., 1.24 ng m^−3^ in the practice gym completed in 2012). 

In the literature, a different distribution of PCBs was reported for workplaces. Manufacturing plants showed high concentrations (709 ng g^−1^), and similar PCB values (range 107–233 ng g^−1^) were observed in schools, offices, electronic factories, hospitals and shopping malls [83]. PCBs were also detected in the air of paper industries, at a concentration up to 2300 ng m^−3^ [102]. Many studies investigated PCB concentrations in the settled dust around the world. For example, PCBs values ranged from 11 to 1900 ng g^−1^ in northern Vietnam [87], while higher concentrations of total PCBs (199–43,540 ng g^−1^) were detected in dwellings and churches in Illinois, US [94]. 

Regarding e-waste recycling sites, higher concentrations were discovered in North-Rhine Westphalia, Germany (from 38,000 to 330,000 ng g^−1^) [97] compared to those in indoor dust from Quingyuan, southern China (568–11,500 ng g^−1^) [86] and in Durban, South Africa (50–490 ng g^−1^) [100]. A recent study reported information on the distribution and composition of PCBs in electronic repair workshop dust in Nigeria. The results indicated concentrations of PCBs from 96.6 to 3949 ng g^−1^ with a mean value of 1234 ng g^−1^ with hexa-PCBs being the most prevalent PCB homologs, which have a high estimated hazard index and cancer risk values associated with human exposure [98]. The regulation of the e-waste problem requires more attention and many efforts, such as source control, limitation of illegal importation of domestic e-waste collection, transportation and process control. 

Environmental remediation measures of such polluted areas should be implemented to control the health risks facing the workers and local population.

### 3.3. Presence of PCB in Human Fluids and Bioaccumulation

Considering the above-mentioned pathways for human exposure to PCBs and their resistance to chemical and thermal degradation, the bioaccumulation of these compounds must be taken into account. 

The bioaccumulation of toxic substances can take place either directly from the environment in which the organism lives (bioconcentration) or through ingestion along the trophic chains (biomagnification or biological magnification) or in both ways. Biomagnification is a process in which a chemical compound accumulates through the food chain from lower concentrations in prey species to higher concentrations in predatory species. In the case of bioconcentration, the amount of substance in the body’s tissues becomes progressively higher than those present in the environment from which it was absorbed. Since PCBs are found in the organic part of the soil and marine and lake sediments, they can be absorbed by plants and ingested by aquatic organisms. Due to their poor degradability, this phenomenon leads to biomagnification along the trophic levels of the food chain. PCBs bioaccumulate in the adipose tissue of living organisms, so their concentration increases along the trophic web, together with their toxicity for both animals and humans. High-chlorine PCBs have a greater potential for bioaccumulation and biomagnification along the food chain [103,104,105].

The biomonitoring approach in blood and non-invasive biological matrices, such as urine, was used to assess worker exposure to PCBs [106]. For instance, concentrations of PCB metabolites up to 174 μg L^−1^ have been detected in human urine from former PCB-exposed workers of a transformer recycling company in Germany [107]. The highest concentrations were observed for low-chlorine PCBs, to which workers are easily exposed through inhalation. Several studies have shown that detection of PCBs in blood is still a serious cause for concern [108,109,110,111,112,113,114]. PCB concentrations up to 0.442 μg L^−1^ were detected in the plasma of a group working in a building with documented PCB contamination (total indoor air PCB concentration in the range 70–1500 ng m^−3^) [115]. Furthermore, data from the last decade demonstrate widespread human exposure to non-legacy PCB congeners that were not present in commercial PCB blends [13]. Non-legacy PCBs were also detected. In particular, PCB 11, one of the most frequently detected PCBs [116], was found at a concentration from 0.005 to 1.717 μg L^−1^ in the plasma of pregnant women. Along with it, different dioxin-like PCBs inadvertently formed as by-products in chemical processes, have also been found at high concentrations. Despite the evidence of the widespread presence of non-legacy PCBs in the environment and consumer products, the metabolism of PCBs and the physiological fate of individual metabolites remain poorly understood.

## 4. Effects of PCBs on Human Health

**Figure 3:**
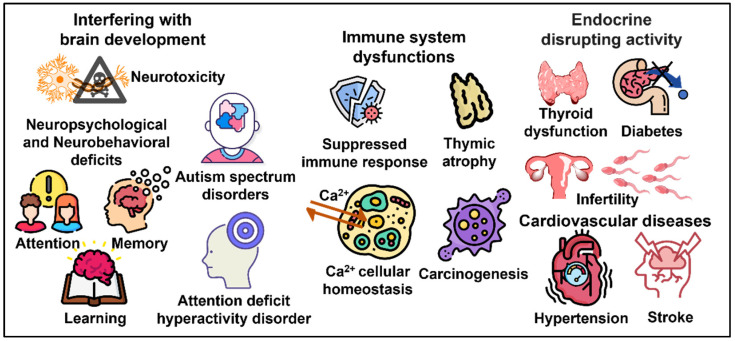
Representation of the main targets of PCBs with related disorders in humans.

The International Agency for Research on Cancer (IARC) has classified PCBs as probable human carcinogens (Group 2A) [117]. PCBs target several human systems, including the nervous system, the endocrine systems (thyroid, thymus, pancreas and gonads), the reproductive system, the cardiovascular system and the immune system (Figure 3). This review will focus in particular on neurological and reproductive health outcomes.

### 4.1. Nervous System Disorders and Other Dysfunctions

The developing brain was identified as a vulnerable target for PCBs by different scientific studies on both humans and animals [118]. Numerous reviews of the epidemiological literature have inferred that exposure to PCBs during nervous system development enhances the risks of neuropsychological deficits in children, as demonstrated by impaired cognitive and psychomotor function, as well as attention, learning and memory deficits [119,120,121,122]. Moreover, recent studies suggest that prenatal exposure to PCBs may increase the risk of autism spectrum disorders [120,123,124,125,126,127] and attention deficit hyperactivity disorder [128,129,130,131]. Prenatal exposure to PCBs is also associated with an increased risk of low birth weight, defined as <2500 g at birth, [132,133,134,135,136] and lower development for gestational age [137,138,139]. Experimental studies on animals confirm that PCB exposures cause neurobehavioral effects similar to those observed in humans [140,141,142,143]. Recent studies suggested that PCB 11 is able to alter the dendritic and axonal growth of neurons by interfering with brain development [144]. However, the 209 PCB congeners that are capable of producing neurotoxic effects and the mechanisms by which PCBs interfere with nervous system development still remain to be determined. In addition, new questions are emerging about the potential neurotoxicity of low-chlorine PCBs, not only those released from PCB-containing equipment and materials manufactured before the production ban, but also the non-legacy PCBs that represent a significant proportion of contemporary human PCB exposures. Recent epidemiological studies suggest that non-dioxin-like PCBs and low-chlorine PCBs are primarily responsible for PCB-associated neurotoxicity [118]. On the other hand, dioxin-like PCBs are associated with diseases that affect various organs; in particular, the skin, liver and immune system [145,146,147] are also carcinogenic [148,149,150,151,152,153,154,155,156].

Since PCB serum levels have been linked to chronic diseases, their possible association with the incidence of all-cause dementia and Parkinson’s disease was also assessed using a population-based prospective cohort study in a north Italian highly polluted area [157]. A positive association between the onset of dementia not mediated by hypertension and total PCB serum levels was observed, whereas the unstable risk estimates for Parkinson’s disease did not permit to conclude a possible association. PCBs have been suspected for some time of having adverse effects on neuropsychological functioning in humans and there are studies that have found associations between serum PCB levels and neurobehavioral deficits in older adults; while there is evidence of slowing of cognitive function in children associated with exposure to PCBs, the evidence of comparable effects on adults is far less well understood [158,159,160].

The results of different epidemiological studies indicate that exposure to PCBs is also associated with immune system dysfunctions, including thymic atrophy and suppressed immune responses, [161,162,163] and cardiovascular diseases, such as stroke and hypertension [164,165,166,167,168,169]. In addition, several studies have shown that non-dioxine-like PCBs alter the cellular homeostasis of calcium, increasing the levels of intracellular Ca^2+^ ions and/or the activation of different cellular processes mediated by the same Ca^2+^ ions. In fact, they operate by altering the structure and function of ryanodine receptors, channels that allow the release of calcium from the sarcoplasmic reticulum of muscle cells and from the endoplasmic reticulum, which is found in other cells [170,171]. Furthermore, the mechanism of PCB toxicity comprises the inhibition of antioxidant defense enzymes, including superoxide dismutase, catalase, glutathione peroxidase, glutathione reductase and glutathione transferase [172,173,174,175,176,177].

### 4.2. Endocrine Disrupting Activity and Effects on Reproductive Organs

PCBs have been considered endocrine disruptors because their exposure was associated with diabetes [46,178], cardiovascular diseases—mainly hypertension [166] and the functionality of the endocrine system, particularly the thyroid and reproductive organs (the primary targets of most endocrine disruptors) [3,179,180]. Several studies have shown that PCBs can interfere with endocrine processes and persistent exposure to them can considerably decrease animal and human fertility and reproductive quality [181]. In fact, the reproductive toxicity of PCBs was proved in both animal and human studies [182,183]. However, their role and mechanism of action are still poorly understood and current evidence is still inconclusive. A study conducted in northern Italy revealed no association between PCB exposure and prevalence of endocrine and metabolic diseases and hypertension [35].

PCBs have been found in follicular and amniotic fluid, uterine muscle, ovarian tissue, placenta, fetal cord blood and breast milk [184,185]. PCBs accumulate over time in human follicular fluid, as observed for the accumulation in serum [186]. Moreover, high PCB concentrations in the follicular fluids are found in women experiencing assisted reproductive technology, which can contribute to in vitro fertilization failure [187]. Women’s exposure to PCBs can damage ovarian function, leading to reproductive problems, such as abnormal hormone levels, premature ovarian failure and finally infertility [188]. Other adverse effects in women, associated with PCB exposure, are the earlier onset of menopause, altered menstrual function, the increase in miscarriage risk and of time taken to get pregnant [189,190,191,192]. In contrast to this evidence, a recent study examined the link between the level of PCBs in serum and various female reproductive health outcomes, finding no correlation between serum PCB levels and infertility, but only an association between the reduction in the number of pregnancies and PCB concentration [193]. Moreover, the majority of the studies that found an association between PCB exposure during pregnancy and reduced cognitive functions among children have not taken into consideration some confounding factors, such as the co-exposure to other toxic compounds (i.e., Hg, Pb and iodine).

Male exposure to endocrine disruptors, such as PCBs and their consequent bioaccumulation, has been associated with the reduction in semen quality, fertility and anogenital distance [34,194,195,196,197,198,199]. In particular, environmental exposure to PCBs influences circulating reproductive hormone levels, sperm concentration, motility, morphology and quantity and quality of gametes, and it alters the redox state of the seminal plasma and other sperm factors (e.g., sperm DNA integrity) [34,194,195,200,201]. A correlation between increasing serum PCB levels and lower concentration of serum testosterone in some American males was noticed [202]. Furthermore, significant associations between low environmental levels of serum dioxin-like PCBs in male partners of subfertile couples and pregnancy outcomes of in vitro fertilization, such as implantation, clinical pregnancy and live births were observed [203]. PCBs may have direct effects on spermatogenesis as they easily penetrate the blood–testis barrier [204], and their accumulation is also associated with testicular cancer [205]. A constant decline in the quality of human semen was observed in many industrialized countries [206]. This probably arises from continuous, repeated and prolonged exposure to POPs that are still today widely used in the production chains of food and consumer products [207,208,209,210]. 

In addition to the effects on the people directly exposed, PCBs might influence the epigenetic modification process since their harmful effects on the reproductive system can be passed to offspring. In fact, PCBs can be transferred from mother to fetus via the placenta [211], resulting in transgenerational effects [212,213], such as heritable epimutations in sperm and brain [214]. Prenatal exposure to PCBs affects gestational length and birth weight [215,216] and causes the reduction in intelligence quotient and fecundity in the offspring [217,218,219]. Moreover, in-utero exposure to PCBs results in children having sperm with abnormal morphology, reduced motility and capacity to penetrate hamster oocytes [220], a reduction in male reproductive function that is transferred on to the next three generations [221]. Not least, high maternal blood concentrations of PCBs at the end of pregnancy are linked to the reduction in anogenital distance in male neonates, a parameter considered as a promising marker of male reproductive health [222].

Although PCBs are indeed declining, as also demonstrated by Raffetti et al. [223], being persistent they are not currently completely absent in various biological fluids. After all, PCBs, used in various types of industrial products, are persistent organochlorine pollutants, considered a potential endocrine disrupting compound, and exposure to these toxins has a negative impact on the chromatin integrity of spermatozoa [224]. Therefore, it cannot be ruled out that these pollutants could have a synergistic action with other pollutants, such as some heavy metals, that have been found in the sperm of subjects residing in areas of high environmental impact. In fact, there are numerous reports demonstrating the correlation between heavy metals with oxidative damage to DNA. In particular, it has been shown that some heavy metals have the potential to alter the properties of the sperm nuclear basic proteins (SNBP) in individuals residing in high environmental impact areas. In these subjects, surprisingly, the SNBP, instead of having their canonical role of protecting DNA, are involved in oxidative DNA damage [225]. In addition, seminal antioxidant activity has also been shown to be lower in these subjects living in areas of high environmental impact [226]. Environmental pollution also has a significant bearing on the susceptibility of a given population to various diseases, and semen quality has been found to be a potential indicator of susceptibility to viral insults in those highly polluted areas, capable of helping to predict the risk of harmful effects of viral outbreaks [227,228]. Very recently it has also been demonstrated that kallikrein-related serine peptidase 3 appears to be an early biomarker of environmental exposure in young women [229]. For these reasons, biosensors have been developed for environmental pollution along with new technologies, especially because altered environmental conditions, together with the direct and indirect short- and long-term effects of viral infection, have the potential to produce a deterioration in sperm quality with significant implications for male fertility, particularly in those areas with a greater environmental impact [230]. Pollutants, such as heavy metals, polycyclic aromatic hydrocarbons, polychlorinated biphenyls, dioxins, pesticides and ultrafine particles, produced by human activities pose real threats to the body’s entire defense system. Trials from preclinical and clinical research studies indicate that compromised male fertility and gonadal development, as well as cancers of the reproductive system, resulting from the exposure to organic and inorganic pollutants can be contrasted by flavonoids [231].

It should be emphasized that the majority of the studies are carried out on adult subjects to verify the possible association of the effects on fertility with ongoing exposures in adult males. However, the exposure of a developing organism may have more pronounced and persistent negative effects. Collectively, these data indicate the need for biological monitoring studies on PCBs taking into account not only the substances widely used, but also paying attention to the potential biomarkers indicative of long-term effects (e.g., sperm DNA damage) and the co-exposure to other toxic compounds.

## 5. Conclusions

In this review, we considered recent scientific studies related to the presence of PCBs in the environment. In particular, we discussed the potential routes for their release into the environment and consequent human exposure, occupational exposure events, and related effects on human health. Dietary intake was the main exposure pathway, even if the contribution from indoor air inhalation could become comparable to dietary exposure in the next years. On the other hand, we have underlined how several literature studies have detected high PCB concentrations in indoor environments (both air and dust) derived from building material (furniture, paints, caulking compounds and sealants) with the consequent transport to the human web. Furthermore, e-waste recycling sites resulted to be the most PCB-contaminated workplaces (concentration up to 330,000 ng g^−1^). These phenomena are of particular concern considering the occupational exposure of workers, indicating the need for better remediation strategies of such polluted workplaces in order to prevent health problems of workers and local populations. Negative effects on human health (neuropsychological and neurobehavioral deficits in children, dementia, immune system dysfunctions, cardiovascular diseases and cancer) were reported to occur at higher PCB concentrations compared to human exposure, demonstrating a hazard for human health. Although PCBs exposure does not necessarily entail clinically relevant consequences in the short term, recent studies suggest that their bioaccumulation can reduce fertility with transgenerational effects. Further studies must be performed to assess the real consequences of PCBs contamination at concentrations in the range of human exposure.

## Figures and Tables

**Table 1:** Concentration of PCB in indoor environments around the world.

Continent	Country	Location	Type of Site	Concentration	Reference
Asia	Taiwan	Tainan	Urban	4.730 ng m^−2^ day^−1^	[80]
			Urban/industrial/rural	0.57–0.65 ng m^−2^ day^−1^	[81]
	South Korea	Pohang	Industrial	2.1 ng m^−2^ day^−1^	[82]
	Japan	Hong Kong	Office	52.5–589 ng g^−1^	[83]
			Electronic factory	47–249 ng g^−1^	
			Manufacturing plan	709 ng g^−1^	
			Electronic factory, commercial office, hospital, school and shopping store	107–233 ng g^−1^	
	China	-	Nonferrous Metallurgical Facilities	0.0155–0.770 ng m^−3^	[84]
		Taizhou	E-waste recycling site	37.75–65.83 ng m^−3^	[85]
			Urban	5.28–21.48 ng m^−3^	
		Quingyuan and Guangzhou	E-waste recycling site	568–11,500 ng g^−1^	[86]
			Rural	55.3–658 ng g^−1^	
			Urban	38.6–226 ng g^−1^	
			Industrial	0.94–1665 ng g^−1^	
	Vietnam	-	Home	11–1900 ng g^−1^	[87]
	Singapore	Singapore	Home	5.6 ng g^−1^	[88]
	India	Chennai	E-waste recycling site	3.6–53 ng g^−1^	[89]
			suburban industrial roadsides	1.6 ng g^−1^	
America	Canada	Toronto	Home	56–820 ng g^−1^	[90]
			Home air	0.11–5.11 ng m^−3^	[17]
			Home dust	<LOD-521 ng g^−1^	
	United States	Chicago	Urban	4500 ng m^−2^ day^−1^	[91]
			Resident	190 ng m^−2^ day^−1^	[92]
			Urban-industrial	0.075–5.5 ng m^−3^	[23]
		New Jersey	Urban	10–40 ng m^−2^ day^−1^	[93]
			Suburban	0.9–3 ng m^−2^ day^−1^	
			Background	0.8–2 ng m^−2^ day^−1^	
		Texas	Home	47–620 ng g^−1^	[90]
		Illinois	Dwelling and church	199–43,540 ng g^−1^	[94]
		Iowa	School	39.2–1.24 ng m^−3^	[95]
		Indiana and Iowa	School	0.5–194 ng m^−3^	[79]
Europe	United Kingdom	Birmingham	Home	57–860 ng g^−1^	[90]
	France	Thau lagoon	Rural	0.715 ng m^−2^ day^−1^	[96]
	Germany	Stuttgart	School	3643–13,561 ng m^−3^	[61]
		North-Rhine Westphalia	E-waste recycling site	8000–330,000 ng g^−1^	[97]
	Czech Republic	Brno	Home air	0.14–4.23 ng m^−3^	[17]
			Home dust	11.4–358 ng g^−1^	
Africa	Nigeria	Abraka and Warri	Office	96.6–3949 ng g^−1^	[98]
		Lagos	Power Station office	0.02–2.20 ng m^−2^ day^−1^	[99]
	South Africa	Durban	E-waste recycling site	50–490 ng g^−1^	[100]
			Office	923–1040 ng g^−1^	
			Computer laboratory	360–1880 ng g^−1^	
Oceania	New Zealand	Wellington	Home	46 ng g^−1^	[90]
-	Turkey	Izmir	Industrial	409 ng m^−2^ day^−1^	[101]

## Data Availability

Not applicable.

## References

[1] Jones K.C., de Voogt P. (1999). Persistent Organic Pollutants (POPs): State of the Science. Environ. Pollut..

[2] Alharbi O.M.L., Basheer A.A., Khattab R.A., Ali I. (2018). Health and Environmental Effects of Persistent Organic Pollutants. J. Mol. Liq..

[3] Pironti C., Ricciardi M., Proto A., Bianco P.M., Montano L., Motta O. (2021). Endocrine-Disrupting Compounds: An Overview on Their Occurrence in the Aquatic Environment and Human Exposure. Water.

[4] Vasseghian Y., Hosseinzadeh S., Khataee A., Dragoi E.-N. (2021). The Concentration of Persistent Organic Pollutants in Water Resources: A Global Systematic Review, Meta-Analysis and Probabilistic Risk Assessment. Sci. Total Environ..

[5] Lallas P.L. (2001). The Stockholm Convention on Persistent Organic Pollutants. Am. J. Int. Law.

[6] Rodrigues J.P., Duarte A.C., Santos-Echeandía J., Rocha-Santos T. (2019). Significance of Interactions between Microplastics and POPs in the Marine Environment: A Critical Overview. TrAC Trends Anal. Chem..

[7] Ricciardi M., Pironti C., Motta O., Miele Y., Proto A., Montano L. (2021). Microplastics in the Aquatic Environment: Occurrence, Persistence, Analysis, and Human Exposure. Water.

[8] Pironti C., Ricciardi M., Motta O., Miele Y., Proto A., Montano L. (2021). Microplastics in the Environment: Intake through the Food Web, Human Exposure and Toxicological Effects. Toxics.

[9] James R.C., Busch H., Tamburro C.H., Roberts S.M., Schell J.D., Harbison R.D. (1993). Polychlorinated Biphenyl Exposure and Human Disease. J. Occup. Environ. Med..

[10] Salhotra A.M., Hodgson E. (2012). Chapter Ten—Human Health Risk Assessment for Contaminated Properties. Progress in Molecular Biology and Translational Science.

[11] Vorkamp K. (2016). An Overlooked Environmental Issue? A Review of the Inadvertent Formation of PCB-11 and Other PCB Congeners and Their Occurrence in Consumer Products and in the Environment. Sci. Total Environ..

[12] Mills III S.A., Thal D.I., Barney J. (2007). A Summary of the 209 PCB Congener Nomenclature. Chemosphere.

[13] Grimm F.A., Hu D., Kania-Korwel I., Lehmler H.-J., Ludewig G., Hornbuckle K.C., Duffel M.W., Bergman Å., Robertson L.W. (2015). Metabolism and Metabolites of Polychlorinated Biphenyls. Crit. Rev. Toxicol..

[14] Hansen L.G. (2001). Identification of Steady State and Episodic PCB Congeners from Multiple Pathway Exposures. PCBs Recent Adv. Environ. Toxicol. Health Eff..

[15] Zhao H.X., Adamcakova-Dodd A., Hu D., Hornbuckle K.C., Just C.L., Robertson L.W., Thorne P.S., Lehmler H.-J. (2010). Development of a Synthetic PCB Mixture Resembling the Average Polychlorinated Biphenyl Profile in Chicago Air. Environ. Int..

[16] Robertson L.W., Ludewig G. (2011). Polychlorinated Biphenyl (PCB) Carcinogenicity with Special Emphasis on Airborne PCBs. Gefahrst Reinhalt Luft.

[17] Audy O., Melymuk L., Venier M., Vojta S., Becanova J., Romanak K., Vykoukalova M., Prokes R., Kukucka P., Diamond M.L. (2018). PCBs and Organochlorine Pesticides in Indoor Environments—A Comparison of Indoor Contamination in Canada and Czech Republic. Chemosphere.

[18] Venier M., Hites R.A. (2010). Time Trend Analysis of Atmospheric POPs Concentrations in the Great Lakes Region Since 1990. Environ. Sci. Technol..

[19] Salamova A., Venier M., Hites R.A. (2015). Revised Temporal Trends of Persistent Organic Pollutant Concentrations in Air around the Great Lakes. Environ. Sci. Technol. Lett..

[20] Venier M., Salamova A., Hites R.A. (2019). How to Distinguish Urban vs. Agricultural Sources of Persistent Organic Pollutants?. Curr. Opin. Environ. Sci. Health.

[21] McFarland V.A., Clarke J.U. (1989). Environmental Occurrence, Abundance, and Potential Toxicity of Polychlorinated Biphenyl Congeners: Considerations for a Congener-Specific Analysis. Environ. Health Perspect..

[22] Wethington D.M., Hornbuckle K.C. (2005). Milwaukee, WI, as a Source of Atmospheric PCBs to Lake Michigan. Environ. Sci. Technol..

[23] Hu D., Lehmler H.-J., Martinez A., Wang K., Hornbuckle K.C. (2010). Atmospheric PCB Congeners across Chicago. Atmos. Environ..

[24] Persoon C., Peters T.M., Kumar N., Hornbuckle K.C. (2010). Spatial Distribution of Airborne Polychlorinated Biphenyls in Cleveland, OH and Chicago, IL. Environ. Sci. Technol..

[25] Haarmann-Stemmann T., Abel J. (2006). The Arylhydrocarbon Receptor Repressor (AhRR): Structure, Expression, and Function. Biol. Chem..

[26] Zhang D., Saktrakulkla P., Tuttle K., Marek R.F., Lehmler H.-J., Wang K., Hornbuckle K.C., Duffel M.W. (2021). Detection and Quantification of Polychlorinated Biphenyl Sulfates in Human Serum. Environ. Sci. Technol..

[27] Klocke C., Sethi S., Lein P.J. (2020). The Developmental Neurotoxicity of Legacy vs. Contemporary Polychlorinated Biphenyls (PCBs): Similarities and Differences. Environ. Sci. Pollut. Res..

[28] Basu I., Arnold K.A., Venier M., Hites R.A. (2009). Partial Pressures of PCB-11 in Air from Several Great Lakes Sites. Environ. Sci. Technol..

[29] Hu D., Hornbuckle K.C. (2010). Inadvertent Polychlorinated Biphenyls in Commercial Paint Pigments. Environ. Sci. Technol..

[30] Rodenburg L.A., Du S., Fennell D.E., Cavallo G.J. (2010). Evidence for Widespread Dechlorination of Polychlorinated Biphenyls in Groundwater, Landfills, and Wastewater Collection Systems. Environ. Sci. Technol..

[31] Grossman E. (2013). Nonlegacy PCBs: Pigment Manufacturing By-Products Get a Second Look. Environ. Health Perspect..

[32] Diamond M.L., Melymuk L., Csiszar S.A., Robson M. (2010). Estimation of PCB Stocks, Emissions, and Urban Fate: Will Our Policies Reduce Concentrations and Exposure?. Environ. Sci. Technol..

[33] Kaw H.Y., Kannan N., de Voogt P. (2017). A Review on Polychlorinated Biphenyls (PCBs) and Polybrominated Diphenyl Ethers (PBDEs) in South Asia with a Focus on Malaysia. Reviews of Environmental Contamination and Toxicology Volume 242.

[34] Meeker J.D., Hauser R. (2010). Exposure to Polychlorinated Biphenyls (PCBs) and Male Reproduction. Syst. Biol. Reprod. Med..

[35] Zani C., Magoni M., Speziani F., Leonardi L., Orizio G., Scarcella C., Gaia A., Donato F. (2019). Polychlorinated Biphenyl Serum Levels, Thyroid Hormones and Endocrine and Metabolic Diseases in People Living in a Highly Polluted Area in North Italy: A Population-Based Study. Heliyon.

[36] Onozuka D., Nakamura Y., Tsuji G., Furue M. (2020). Mortality in Yusho Patients Exposed to Polychlorinated Biphenyls and Polychlorinated Dibenzofurans: A 50-Year Retrospective Cohort Study. Environ. Health.

[37] Lee R.G.M., Coleman P., Jones J.L., Jones K.C., Lohmann R. (2005). Emission Factors and Importance of PCDD/Fs, PCBs, PCNs, PAHs and PM10 from the Domestic Burning of Coal and Wood in the U.K. Environ. Sci. Technol..

[38] Shen J., Yang L., Liu G., Zhao X., Zheng M. (2021). Occurrence, Profiles, and Control of Unintentional POPs in the Steelmaking Industry: A Review. Sci. Total Environ..

[39] Wania F. (2003). Assessing the Potential of Persistent Organic Chemicals for Long-Range Transport and Accumulation in Polar Regions. Environ. Sci. Technol..

[40] Gouin T., Mackay D., Jones K.C., Harner T., Meijer S.N. (2004). Evidence for the “Grasshopper” Effect and Fractionation during Long-Range Atmospheric Transport of Organic Contaminants. Environ. Pollut..

[41] Weitekamp C.A., Phillips L.J., Carlson L.M., DeLuca N.M., Cohen Hubal E.A., Lehmann G.M. (2021). A State-of-the-Science Review of Polychlorinated Biphenyl Exposures at Background Levels: Relative Contributions of Exposure Routes. Sci. Total Environ..

[42] Schecter A., Colacino J., Haffner D., Patel K., Opel M., Päpke O., Birnbaum L. (2010). Perfluorinated Compounds, Polychlorinated Biphenyls, and Organochlorine Pesticide Contamination in Composite Food Samples from Dallas, Texas, USA. Environ. Health Perspect..

[43] Feinberg M., Soler L., Contenot S., Verger P. (2011). Assessment of Seasonality in Exposure to Dioxins, Furans and Dioxin-like PCBs by Using Long-Term Food-Consumption Data. Food Addit. Contam. Part A.

[44] Domingo J.L., Bocio A. (2007). Levels of PCDD/PCDFs and PCBs in Edible Marine Species and Human Intake: A Literature Review. Environ. Int..

[45] Weintraub M., Birnbaum L.S. (2008). Catfish Consumption as a Contributor to Elevated PCB Levels in a Non-Hispanic Black Subpopulation. Environ. Res..

[46] Marushka L., Hu X., Batal M., Tikhonov C., Sadik T., Schwartz H., Ing A., Fediuk K., Chan H.M. (2021). The Relationship between Dietary Exposure to Persistent Organic Pollutants from Fish Consumption and Type 2 Diabetes among First Nations in Canada. Can. J. Public Health.

[47] Lan T., Liu B., Bao W., Thorne P.S. (2021). BMI Modifies the Association between Dietary Intake and Serum Levels of PCBs. Environ. Int..

[48] Saktrakulkla P., Lan T., Hua J., Marek R.F., Thorne P.S., Hornbuckle K.C. (2020). Polychlorinated Biphenyls in Food. Environ. Sci. Technol..

[49] Undeman E., Brown T.N., McLachlan M.S., Wania F. (2018). Who in the World Is Most Exposed to Polychlorinated Biphenyls? Using Models to Identify Highly Exposed Populations. Environ. Res. Lett..

[50] Ludewig G., Lehmann L., Esch H., Robertson L.W. (2008). Metabolic Activation of PCBs to Carcinogens in vivo—A Review. Environ. Toxicol. Pharmacol..

[51] Hombrecher K., Quass U., Leisner J., Wichert M. (2021). Significant Release of Unintentionally Produced Non-Aroclor Polychlorinated Biphenyl (PCB) Congeners PCB 47, PCB 51 and PCB 68 from a Silicone Rubber Production Site in North Rhine-Westphalia, Germany. Chemosphere.

[52] Ockenden W.A., Lohmann R., Shears J.R., Jones K.C. (2001). The Significance of PCBs in the Atmosphere of the Southern Hemisphere. Env. Sci Pollut Res.

[53] Sun P., Basu I., Hites R.A. (2006). Temporal Trends of Polychlorinated Biphenyls in Precipitation and Air at Chicago. Environ. Sci. Technol..

[54] Choi A.L., Levy J.I., Dockery D.W., Ryan L.M., Tolbert P.E., Altshul L.M., Korrick S.A. (2006). Does Living Near a Superfund Site Contribute to Higher Polychlorinated Biphenyl (PCB) Exposure?. Environ. Health Perspect..

[55] Breivik K., Sweetman A., Pacyna J.M., Jones K.C. (2007). Towards a Global Historical Emission Inventory for Selected PCB Congeners—A Mass Balance Approach: 3. An Update. Sci. Total Environ..

[56] Melymuk L., Robson M., Helm P.A., Diamond M.L. (2012). PCBs, PBDEs, and PAHs in Toronto Air: Spatial and Seasonal Trends and Implications for Contaminant Transport. Sci. Total Environ..

[57] US EPA Great Lakes Integrated Atmospheric Deposition Network. https://www.epa.gov/great-lakes-monitoring/great-lakes-integrated-atmospheric-deposition-network.

[58] Rudel R.A., Perovich L.J. (2009). Endocrine Disrupting Chemicals in Indoor and Outdoor Air. Atmos. Environ..

[59] Choi S.-D., Baek S.-Y., Chang Y.-S., Wania F., Ikonomou M.G., Yoon Y.-J., Park B.-K., Hong S. (2008). Passive Air Sampling of Polychlorinated Biphenyls and Organochlorine Pesticides at the Korean Arctic and Antarctic Research Stations: Implications for Long-Range Transport and Local Pollution. Environ. Sci. Technol..

[60] Anh H.Q., Watanabe I., Minh T.B., Takahashi S. (2021). Unintentionally Produced Polychlorinated Biphenyls in Pigments: An Updated Review on Their Formation, Emission Sources, Contamination Status, and Toxic Effects. Sci. Total Environ..

[61] Gabrio T., Piechotowski I., Wallenhorst T., Klett M., Cott L., Friebel P., Link B., Schwenk M. (2000). PCB-Blood Levels in Teachers, Working in PCB-Contaminated Schools. Chemosphere.

[62] Schwenk M., Gabrio T., Päpke O., Wallenhorst T. (2002). Human Biomonitoring of Polychlorinated Biphenyls and Polychlorinated Dibenzodioxins and Dibenzofuranes in Teachers Working in a PCB-Contaminated School. Chemosphere.

[63] Liebl B., Schettgen T., Kerscher G., Broding H.-C., Otto A., Angerer J., Drexler H. (2004). Evidence for Increased Internal Exposure to Lower Chlorinated Polychlorinated Biphenyls (PCB) in Pupils Attending a Contaminated School. Int. J. Hyg. Environ. Health.

[64] Fitzgerald E.F., Shrestha S., Palmer P.M., Wilson L.R., Belanger E.E., Gomez M.I., Cayo M.R., Hwang S. (2011). Polychlorinated Biphenyls (PCBs) in Indoor Air and in Serum among Older Residents of Upper Hudson River Communities. Chemosphere.

[65] Hu D., Martinez A., Hornbuckle K.C. (2008). Discovery of Non-Aroclor PCB (3,3′-Dichlorobiphenyl) in Chicago Air. Environ. Sci. Technol..

[66] Wolff M.S. (1985). Occupational Exposure to Polychlorinated Biphenyls (PCBs). Environ. Health Perspect..

[67] Ross G. (2004). The Public Health Implications of Polychlorinated Biphenyls (PCBs) in the Environment. Ecotoxicol. Environ. Saf..

[68] Pironti C., Ricciardi M., Proto A., Cucciniello R., Fiorentino A., Fiorillo R., Motta O. (2022). New Analytical Approach to Monitoring Air Quality in Historical Monuments through the Isotopic Ratio of CO_2_. Environ. Sci. Pollut. Res..

[69] Motta O., Pironti C., Ricciardi M., Rostagno C., Bolzacchini E., Ferrero L., Cucciniello R., Proto A. (2022). Leonardo Da Vinci’s “Last Supper”: A Case Study to Evaluate the Influence of Visitors on the Museum Preservation Systems. Environ. Sci. Pollut. Res..

[70] Ricciardi M., Pironti C., Motta O., Fiorillo R., Camin F., Faggiano A., Proto A. (2022). Investigations on Historical Monuments’ Deterioration through Chemical and Isotopic Analyses: An Italian Case Study. Envron. Sci. Pollut. Res..

[71] ATSDR (2000). Toxicological Profile for Polychlorinated Biphenyls (Pcbs).

[72] Kohler M., Tremp J., Zennegg M., Seiler C., Minder-Kohler S., Beck M., Lienemann P., Wegmann L., Schmid P. (2005). Joint Sealants:  An Overlooked Diffuse Source of Polychlorinated Biphenyls in Buildings. Environ. Sci. Technol..

[73] Herrick R.F., McClean M.D., Meeker J.D., Baxter L.K., Weymouth G.A. (2004). An Unrecognized Source of PCB Contamination in Schools and Other Buildings. Environ. Health Perspect..

[74] Jamshidi A., Hunter S., Hazrati S., Harrad S. (2007). Concentrations and Chiral Signatures of Polychlorinated Biphenyls in Outdoor and Indoor Air and Soil in a Major U.K. Conurbation. Environ. Sci. Technol..

[75] Herrick R.F. (2010). PCBs in School—Persistent Chemicals, Persistent Problems. New Solut..

[76] Harrad S., Goosey E., Desborough J., Abdallah M.A.-E., Roosens L., Covaci A. (2010). Dust from U.K. Primary School Classrooms and Daycare Centers: The Significance of Dust As a Pathway of Exposure of Young U.K. Children to Brominated Flame Retardants and Polychlorinated Biphenyls. Environ. Sci. Technol..

[77] Zhang X., Diamond M.L., Robson M., Harrad S. (2011). Sources, Emissions, and Fate of Polybrominated Diphenyl Ethers and Polychlorinated Biphenyls Indoors in Toronto, Canada. Environ. Sci. Technol..

[78] MacIntosh D.L., Minegishi T., Fragala M.A., Allen J.G., Coghlan K.M., Stewart J.H., McCarthy J.F. (2012). Mitigation of Building-Related Polychlorinated Biphenyls in Indoor Air of a School. Environ. Health.

[79] Marek R.F., Thorne P.S., Herkert N.J., Awad A.M., Hornbuckle K.C. (2017). Airborne PCBs and OH-PCBs Inside and Outside Urban and Rural U.S. Schools. Environ. Sci. Technol..

[80] Lee W.-J., Su C.-C., Sheu H.-L., Fan Y.-C., Chao H.-R., Fang G.-C. (1996). Monitoring and Modeling of PCB Dry Deposition in Urban Area. J. Hazard. Mater..

[81] Mi H.-H., Wu Z.-S., Lin L.-F., Lai Y.-C., Lee Y.-Y., Wang L.-C., Chang-Chien G.-P. (2012). Atmospheric Dry Deposition of Polychlorinated Dibenzo-p-Dioxins/Dibenzofurans (PCDD/Fs) and Polychlorinated Biphenyls (PCBs) in Southern Taiwan. Aerosol Air Qual. Res..

[82] Fang M., Choi S.-D., Baek S.-Y., Jin G., Chang Y.-S. (2012). Deposition of Polychlorinated Biphenyls and Polybrominated Diphenyl Ethers in the Vicinity of a Steel Manufacturing Plant. Atmos. Environ..

[83] Kang Y., Yin Y., Man Y., Li L., Zhang Q., Zeng L., Luo J., Wong M.H. (2013). Bioaccessibility of Polychlorinated Biphenyls in Workplace Dust and Its Implication for Risk Assessment. Chemosphere.

[84] Hu J., Zheng M., Liu W., Li C., Nie Z., Liu G., Xiao K., Dong S. (2013). Occupational Exposure to Polychlorinated Dibenzo-p-Dioxins and Dibenzofurans, Dioxin-like Polychlorinated Biphenyls, and Polychlorinated Naphthalenes in Workplaces of Secondary Nonferrous Metallurgical Facilities in China. Environ. Sci. Technol..

[85] Wang Y., Hu J., Lin W., Wang N., Li C., Luo P., Hashmi M.Z., Wang W., Su X., Chen C. (2016). Health Risk Assessment of Migrant Workers’ Exposure to Polychlorinated Biphenyls in Air and Dust in an e-Waste Recycling Area in China: Indication for a New Wealth Gap in Environmental Rights. Environ. Int..

[86] He C.-T., Zheng X.-B., Yan X., Zheng J., Wang M.-H., Tan X., Qiao L., Chen S.-J., Yang Z.-Y., Mai B.-X. (2017). Organic Contaminants and Heavy Metals in Indoor Dust from E-Waste Recycling, Rural, and Urban Areas in South China: Spatial Characteristics and Implications for Human Exposure. Ecotoxicol. Environ. Saf..

[87] Anh H.Q., Watanabe I., Minh T.B., Tue N.M., Tuyen L.H., Viet P.H., Takahashi S. (2020). Polychlorinated Biphenyls in Settled Dusts from an End-of-Life Vehicle Processing Area and Normal House Dusts in Northern Vietnam: Occurrence, Potential Sources, and Risk Assessment. Sci. Total Environ..

[88] Tan J., Cheng S.M., Loganath A., Chong Y.S., Obbard J.P. (2007). Selected Organochlorine Pesticide and Polychlorinated Biphenyl Residues in House Dust in Singapore. Chemosphere.

[89] Chakraborty P., Prithiviraj B., Selvaraj S., Kumar B. (2016). Polychlorinated Biphenyls in Settled Dust from Informal Electronic Waste Recycling Workshops and Nearby Highways in Urban Centers and Suburban Industrial Roadsides of Chennai City, India: Levels, Congener Profiles and Exposure Assessment. Sci. Total Environ..

[90] Harrad S., Ibarra C., Robson M., Melymuk L., Zhang X., Diamond M., Douwes J. (2009). Polychlorinated Biphenyls in Domestic Dust from Canada, New Zealand, United Kingdom and United States: Implications for Human Exposure. Chemosphere.

[91] Holsen T.M., Noll K.E., Liu S.P., Lee W.J. (1991). Dry Deposition of Polychlorinated Biphenyls in Urban Areas. Environ. Sci. Technol..

[92] Tasdemir Y., Vardar N., Odabasi M., Holsen T.M. (2004). Concentrations and Gas/Particle Partitioning of PCBs in Chicago. Environ. Pollut..

[93] Van Ry D.A., Gigliotti C.L., Glenn, Nelson E.D., Totten L.A., Eisenreich S.J. (2002). Wet Deposition of Polychlorinated Biphenyls in Urban and Background Areas of the Mid-Atlantic States. Environ. Sci. Technol..

[94] Gonzalez J., Feng L., Sutherland A., Waller C., Sok H., Hesse P.R., Rosenfeld P.D.P. (2011). PCBs and Dioxins/Furans in Attic Dust Collected near Former PCB Production and Secondary Copper Facilities in Sauget, IL. Procedia Environ. Sci..

[95] Bannavti M.K., Jahnke J.C., Marek R.F., Just C.L., Hornbuckle K.C. (2021). Room-to-Room Variability of Airborne Polychlorinated Biphenyls in Schools and the Application of Air Sampling for Targeted Source Evaluation. Environ. Sci. Technol..

[96] Castro-Jiménez J., Mariani G., Vives I., Skejo H., Umlauf G., Zaldívar J.M., Dueri S., Messiaen G., Laugier T. (2011). Atmospheric Concentrations, Occurrence and Deposition of Persistent Organic Pollutants (POPs) in a Mediterranean Coastal Site (Etang de Thau, France). Environ. Pollut..

[97] Klees M., Hombrecher K., Gladtke D. (2017). Polychlorinated Biphenyls in the Surrounding of an E-Waste Recycling Facility in North-Rhine Westphalia: Levels in Plants and Dusts, Spatial Distribution, Homologue Pattern and Source Identification Using the Combination of Plants and Wind Direction Data. Sci. Total Environ..

[98] Iwegbue C.M.A., Eyengho S.B., Egobueze F.E., Odali E.W., Tesi G.O., Nwajei G.E., Martincigh B.S. (2019). Polybrominated Diphenyl Ethers and Polychlorinated Biphenyls in Indoor Dust from Electronic Repair Workshops in Southern Nigeria: Implications for Onsite Human Exposure. Sci. Total Environ..

[99] Abafe O.A., Martincigh B.S. (2015). Polybrominated Diphenyl Ethers and Polychlorinated Biphenyls in Indoor Dust in Durban, South Africa. Indoor Air.

[100] Abafe O.A., Martincigh B.S. (2015). An Assessment of Polybrominated Diphenyl Ethers and Polychlorinated Biphenyls in the Indoor Dust of E-Waste Recycling Facilities in South Africa: Implications for Occupational Exposure. Environ. Sci. Pollut. Res..

[101] Bozlaker A., Odabasi M., Muezzinoglu A. (2008). Dry Deposition and Soil–Air Gas Exchange of Polychlorinated Biphenyls (PCBs) in an Industrial Area. Environ. Pollut..

[102] Korhonen K., Liukkonen T., Ahrens W., Astrakianakis G., Boffetta P., Burdorf A., Heederik D., Kauppinen T., Kogevinas M., Osvoll P. (2004). Occupational Exposure to Chemical Agents in the Paper Industry. Int. Arch. Occup. Environ. Health.

[103] Barron M.G., Yurk J.J., Crothers D.B. (1994). Assessment of Potential Cancer Risk from Consumption of PCBs Bioaccumulated in Fish and Shellfish. Environ. Health Perspect..

[104] Troisi G.M., Haraguchi K., Kaydoo D.S., Nyman M., Aguilar A., Borrell A., Siebert U., Mason C.F. (2000). Bioaccumulation of Polychlorinated Biphenyls (PCBs) and Dichlorodiphenylethane (DDE) Methyl Sulfones in Tissues of Seal and Dolphin Morbillivirus Epizootic Victims. J. Toxicol. Environ. Health Part A.

[105] Oregel-Zamudio E., Alvarez-Bernal D., Franco-Hernandez M.O., Buelna-Osben H.R., Mora M. (2021). Bioaccumulation of PCBs and PBDEs in Fish from a Tropical Lake Chapala, Mexico. Toxics.

[106] Link B., Gabrio T., Zoellner I., Piechotowski I., Paepke O., Herrmann T., Felder-Kennel A., Maisner V., Schick K.-H., Schrimpf M. (2005). Biomonitoring of Persistent Organochlorine Pesticides, PCDD/PCDFs and Dioxin-like PCBs in Blood of Children from South West Germany (Baden-Wuerttemberg) from 1993 to 2003. Chemosphere.

[107] Quinete N., Esser A., Kraus T., Schettgen T. (2016). Determination of Hydroxylated Polychlorinated Biphenyls (OH-PCBs) in Human Urine in a Highly Occupationally Exposed German Cohort: New Prospects for Urinary Biomarkers of PCB Exposure. Environ. Int..

[108] Mari M., Schuhmacher M., Domingo J.L. (2009). Levels of Metals and Organic Substances in Workers at a Hazardous Waste Incinerator: A Follow-up Study. Int. Arch. Occup. Environ. Health.

[109] Hopf N.B., Ruder A.M., Succop P. (2009). Background Levels of Polychlorinated Biphenyls in the U.S. Population. Sci. Total Environ..

[110] Consonni D., Sindaco R., Bertazzi P.A. (2012). Blood Levels of Dioxins, Furans, Dioxin-like PCBs, and TEQs in General Populations: A Review, 1989–2010. Environ. Int..

[111] Nakamoto M., Arisawa K., Uemura H., Katsuura S., Takami H., Sawachika F., Yamaguchi M., Juta T., Sakai T., Toda E. (2013). Association between Blood Levels of PCDDs/PCDFs/Dioxin-like PCBs and History of Allergic and Other Diseases in the Japanese Population. Int. Arch. Occup. Environ. Health.

[112] Koh W.X., Hornbuckle K.C., Thorne P.S. (2015). Human Serum from Urban and Rural Adolescents and Their Mothers Shows Exposure to Polychlorinated Biphenyls Not Found in Commercial Mixtures. Environ. Sci. Technol..

[113] ‘t Mannetje A., Eng A., Walls C., Dryson E., McLean D., Kogevinas M., Fowles J., Borman B., O’Connor P., Cheng S. (2016). Serum Concentrations of Chlorinated Dibenzo-p-Dioxins, Furans and PCBs, among Former Phenoxy Herbicide Production Workers and Firefighters in New Zealand. Int. Arch. Occup. Environ. Health.

[114] Peper M., Klett M., Morgenstern R. (2005). Neuropsychological Effects of Chronic Low-Dose Exposure to Polychlorinated Biphenyls (PCBs): A Cross-Sectional Study. Environ. Health.

[115] Pedersen E.B., Ebbehøj N.E., Göen T., Meyer H.W., Jacobsen P. (2016). Exposure to 27 Polychlorinated Biphenyls in the Indoor Environment of a Workplace: A Controlled Bio-Monitoring Study. Int. Arch. Occup. Environ. Health.

[116] Pizzini S., Sbicego C., Corami F., Grotti M., Magi E., Bonato T., Cozzi G., Barbante C., Piazza R. (2017). 3,3′-Dichlorobiphenyl (Non-Aroclor PCB-11) as a Marker of Non-Legacy PCB Contamination in Marine Species: Comparison between Antarctic and Mediterranean Bivalves. Chemosphere.

[117] IARC (2012). IARC Monographs on the Evaluation of Carcinogenic Risks to Humans.

[118] Klocke C., Lein P.J. (2020). Evidence Implicating Non-Dioxin-Like Congeners as the Key Mediators of Polychlorinated Biphenyl (PCB) Developmental Neurotoxicity. Int. J. Mol. Sci..

[119] Schantz S.L., Widholm J.J., Rice D.C. (2003). Effects of PCB Exposure on Neuropsychological Function in Children. Environ. Health Perspect..

[120] Boucher O., Muckle G., Bastien C.H. (2009). Prenatal Exposure to Polychlorinated Biphenyls: A Neuropsychologic Analysis. Environ. Health Perspect..

[121] Berghuis S.A., Bos A.F., Sauer P.J., Roze E. (2015). Developmental Neurotoxicity of Persistent Organic Pollutants: An Update on Childhood Outcome. Arch. Toxicol..

[122] Pessah I.N., Lein P.J., Seegal R.F., Sagiv S.K. (2019). Neurotoxicity of Polychlorinated Biphenyls and Related Organohalogens. Acta Neuropathol..

[123] Stewart P.W., Lonky E., Reihman J., Pagano J., Gump B.B., Darvill T. (2008). The Relationship between Prenatal PCB Exposure and Intelligence (IQ) in 9-Year-Old Children. Environ. Health Perspect..

[124] Tatsuta N., Nakai K., Murata K., Suzuki K., Iwai-Shimada M., Kurokawa N., Hosokawa T., Satoh H. (2014). Impacts of Prenatal Exposures to Polychlorinated Biphenyls, Methylmercury, and Lead on Intellectual Ability of 42-Month-Old Children in Japan. Environ. Res..

[125] Kyriklaki A., Vafeiadi M., Kampouri M., Koutra K., Roumeliotaki T., Chalkiadaki G., Anousaki D., Rantakokko P., Kiviranta H., Fthenou E. (2016). Prenatal Exposure to Persistent Organic Pollutants in Association with Offspring Neuropsychological Development at 4years of Age: The Rhea Mother-Child Cohort, Crete, Greece. Environ. Int..

[126] Ikeno T., Miyashita C., Nakajima S., Kobayashi S., Yamazaki K., Saijo Y., Kita T., Sasaki S., Konishi K., Kajiwara J. (2018). Effects of Low-Level Prenatal Exposure to Dioxins on Cognitive Development in Japanese Children at 42months. Sci. Total Environ..

[127] Panesar H.K., Kennedy C.L., Keil Stietz K.P., Lein P.J. (2020). Polychlorinated Biphenyls (PCBs): Risk Factors for Autism Spectrum Disorder?. Toxics.

[128] Sagiv S.K., Thurston S.W., Bellinger D.C., Tolbert P.E., Altshul L.M., Korrick S.A. (2010). Prenatal Organochlorine Exposure and Behaviors Associated With Attention Deficit Hyperactivity Disorder in School-Aged Children. Am. J. Epidemiol..

[129] Eubig P.A., Aguiar A., Schantz S.L. (2010). Lead and PCBs as Risk Factors for Attention Deficit/Hyperactivity Disorder. Environ. Health Perspect..

[130] de Cock M., Maas Y.G.H., van de Bor M. (2012). Does Perinatal Exposure to Endocrine Disruptors Induce Autism Spectrum and Attention Deficit Hyperactivity Disorders? Review. Acta Paediatr..

[131] Rosenquist A.H., Høyer B.B., Julvez J., Sunyer J., Pedersen H.S., Lenters V., Jönsson B.A.G., Bonde J.P., Toft G. (2017). Prenatal and Postnatal PCB-153 and p,p′-DDE Exposures and Behavior Scores at 5–9 Years of Age among Children in Greenland and Ukraine. Environ. Health Perspect..

[132] Taylor P.R., Stelma J.M., Lawrence C.J. (1989). The Relation of Polychlorinated Biphenyls to Birth Weight and Gestational Age in the Offspring of Occupationally Exposed Mothers. Am. J. Epidemiol..

[133] Patandin S., Koopman-Esseboom C., De Ridder M.A.J., Weisglas-Kuperus N., Sauer P.J.J. (1998). Effects of Environmental Exposure to Polychlorinated Biphenyls and Dioxins on Birth Size and Growth in Dutch Children. Pediatr. Res..

[134] Baibergenova A., Kudyakov R., Zdeb M., Carpenter D.O. (2003). Low Birth Weight and Residential Proximity to PCB-Contaminated Waste Sites. Environ. Health Perspect..

[135] Hertz-Picciotto I., Charles M.J., James R.A., Keller J.A., Willman E., Teplin S. (2005). In Utero Polychlorinated Biphenyl Exposures in Relation to Fetal and Early Childhood Growth. Epidemiology.

[136] Govarts E., Nieuwenhuijsen M., Schoeters G., Ballester F., Bloemen K., de Boer M., Chevrier C., Eggesbø M., Guxens M., Krämer U. (2012). Birth Weight and Prenatal Exposure to Polychlorinated Biphenyls (PCBs) and Dichlorodiphenyldichloroethylene (DDE): A Meta-Analysis within 12 European Birth Cohorts. Environ. Health Perspect..

[137] Longnecker M.P., Klebanoff M.A., Brock J.W., Guo X. (2005). Maternal Levels of Polychlorinated Biphenyls in Relation to Preterm and Small-for-Gestational-Age Birth. Epidemiology.

[138] Lauritzen H.B., Larose T.L., Øien T., Sandanger T.M., Odland J.Ø., van de Bor M., Jacobsen G.W. (2017). Maternal Serum Levels of Perfluoroalkyl Substances and Organochlorines and Indices of Fetal Growth: A Scandinavian Case—Cohort Study. Pediatr Res.

[139] Govarts E., Iszatt N., Trnovec T., de Cock M., Eggesbø M., Palkovicova Murinova L., van de Bor M., Guxens M., Chevrier C., Koppen G. (2018). Prenatal Exposure to Endocrine Disrupting Chemicals and Risk of Being Born Small for Gestational Age: Pooled Analysis of Seven European Birth Cohorts. Environ. Int..

[140] Sable H.J.K., Schantz S.L. (2006). Executive Function Following Developmental Exposure to Polychlorinated Biphenyls (PCBs): What Animal Models Have Told Us.

[141] Yang D., Kim K.H., Phimister A., Bachstetter A.D., Ward T.R., Stackman R.W., Mervis R.F., Wisniewski A.B., Klein S.L., Kodavanti P.R.S. (2009). Developmental Exposure to Polychlorinated Biphenyls Interferes with Experience-Dependent Dendritic Plasticity and Ryanodine Receptor Expression in Weanling Rats. Environ. Health Perspect..

[142] Winneke G. (2011). Developmental Aspects of Environmental Neurotoxicology: Lessons from Lead and Polychlorinated Biphenyls. J. Neurol. Sci..

[143] Gore A.C., Krishnan K., Reilly M.P. (2019). Endocrine-Disrupting Chemicals: Effects on Neuroendocrine Systems and the Neurobiology of Social Behavior. Horm. Behav..

[144] Sethi S., Keil K.P., Chen H., Hayakawa K., Li X., Lin Y., Lehmler H.-J., Puschner B., Lein P.J. (2017). Detection of 3,3′-Dichlorobiphenyl in Human Maternal Plasma and Its Effects on Axonal and Dendritic Growth in Primary Rat Neurons. Toxicol. Sci..

[145] Mellor C.L., Steinmetz F.P., Cronin M.T.D. (2016). The Identification of Nuclear Receptors Associated with Hepatic Steatosis to Develop and Extend Adverse Outcome Pathways. Crit. Rev. Toxicol..

[146] Bock K.W. (2016). Toward Elucidation of Dioxin-Mediated Chloracne and Ah Receptor Functions. Biochem. Pharmacol..

[147] Wheeler M.A., Rothhammer V., Quintana F.J. (2017). Control of Immune-Mediated Pathology via the Aryl Hydrocarbon Receptor. J. Biol. Chem..

[148] Silberhorn E.M., Glauert H.P., Robertson L.W. (1990). Critical Reviews in: Carcinogenicity of Polyhalogenated Biphenyls: PCBs and PBBs. Crit. Rev. Toxicol..

[149] Loomis D., Browning S.R., Schenck A.P., Gregory E., Savitz D.A. (1997). Cancer Mortality among Electric Utility Workers Exposed to Polychlorinated Biphenyls. Occup. Environ. Med..

[150] National Toxicology Program (2006). NTP Toxicology and Carcinogenesis Studies of 3,3’,4,4’,5-Pentachlorobiphenyl (PCB 126) (CAS No. 57465-28-8) in Female Harlan Sprague-Dawley Rats (Gavage Studies). Natl. Toxicol. Program Tech. Rep. Ser..

[151] National Toxicology Program (2010). Toxicology and Carcinogenesis Studies of 2,3’,4,4’,5-Pentachlorobiphenyl (PCB 118) (CAS No. 31508-00-6) in Female Harlan Sprague-Dawley Rats (Gavage Studies). Natl. Toxicol. Program Tech. Rep. Ser..

[152] Silver S.R., Whelan E.A., Deddens J.A., Steenland N.K., Hopf N.B., Waters M.A., Ruder A.M., Prince M.M., Yong L.C., Hein M.J. (2009). Occupational Exposure to Polychlorinated Biphenyls and Risk of Breast Cancer. Environ. Health Perspect..

[153] Luecke S., Backlund M., Jux B., Esser C., Krutmann J., Rannug A. (2010). The Aryl Hydrocarbon Receptor (AHR), a Novel Regulator of Human Melanogenesis. Pigment. Cell Melanoma Res..

[154] Gallagher R.P., MacArthur A.C., Lee T.K., Weber J.-P., Leblanc A., Mark Elwood J., Borugian M., Abanto Z., Spinelli J.J. (2011). Plasma Levels of Polychlorinated Biphenyls and Risk of Cutaneous Malignant Melanoma: A Preliminary Study. Int. J. Cancer.

[155] Lauby-Secretan B., Loomis D., Grosse Y., El Ghissassi F., Bouvard V., Benbrahim-Tallaa L., Guha N., Baan R., Mattock H., Straif K. (2013). Carcinogenicity of Polychlorinated Biphenyls and Polybrominated Biphenyls. Lancet Oncol..

[156] Ludewig G., Robertson L.W. (2013). Polychlorinated Biphenyls (PCBs) as Initiating Agents in Hepatocellular Carcinoma. Cancer Lett..

[157] Raffetti E., Donato F., De Palma G., Leonardi L., Sileo C., Magoni M. (2020). Polychlorinated Biphenyls (PCBs) and Risk of Dementia and Parkinson Disease: A Population-Based Cohort Study in a North Italian Highly Polluted Area. Chemosphere.

[158] Schantz S.L., Gasior D.M., Polverejan E., McCaffrey R.J., Sweeney A.M., Humphrey H.E., Gardiner J.C. (2001). Impairments of Memory and Learning in Older Adults Exposed to Polychlorinated Biphenyls via Consumption of Great Lakes Fish. Environ. Health Perspect..

[159] Fitzgerald E.F., Belanger E.E., Gomez M.I., Hwang S., Jansing R.L., Hicks H.E. (2007). Environmental Exposures to Polychlorinated Biphenyls (PCBs) among Older Residents of Upper Hudson River Communities. Environ. Res..

[160] Haase R.F., McCaffrey R.J., Santiago-Rivera A.L., Morse G.S., Tarbell A. (2009). Evidence of an Age-Related Threshold Effect of Polychlorinated Biphenyls (PCBs) on Neuropsychological Functioning in a Native American Population. Environ. Res..

[161] Heilmann C., Grandjean P., Weihe P., Nielsen F., Budtz-Jørgensen E. (2006). Reduced Antibody Responses to Vaccinations in Children Exposed to Polychlorinated Biphenyls. PLoS Med..

[162] Selgrade M.K. (2007). Immunotoxicity—The Risk Is Real. Toxicol. Sci..

[163] Park H.-Y., Hertz-Picciotto I., Petrik J., Palkovicova L., Kocan A., Trnovec T. (2008). Prenatal PCB Exposure and Thymus Size at Birth in Neonates in Eastern Slovakia. Environ. Health Perspect..

[164] Hennig B., Reiterer G., Majkova Z., Oesterling E., Meerarani P., Toborek M. (2005). Modification of Environmental Toxicity by Nutrients. Cardiovasc. Toxicol..

[165] Dziennis S., Yang D., Cheng J., Anderson K.A., Alkayed N.J., Hurn P.D., Lein P.J. (2008). Developmental Exposure to Polychlorinated Biphenyls Influences Stroke Outcome in Adult Rats. Environ. Health Perspect..

[166] Everett C.J., Mainous A.G., Frithsen I.L., Player M.S., Matheson E.M. (2008). Association of Polychlorinated Biphenyls with Hypertension in the 1999–2002 National Health and Nutrition Examination Survey. Environ. Res..

[167] Humblet O., Birnbaum L., Rimm E., Mittleman M.A., Hauser R. (2008). Dioxins and Cardiovascular Disease Mortality. Environ. Health Perspect..

[168] Helyar S.G., Patel B., Headington K., El Assal M., Chatterjee P.K., Pacher P., Mabley J.G. (2009). PCB-Induced Endothelial Cell Dysfunction: Role of Poly(ADP-Ribose) Polymerase. Biochem. Pharmacol..

[169] Raffetti E., Donato F., De Palma G., Leonardi L., Sileo C., Magoni M. (2020). Polychlorinated Biphenyls (PCBs) and Risk of Hypertension: A Population-Based Cohort Study in a North Italian Highly Polluted Area. Sci. Total Environ..

[170] Samsó M., Feng W., Pessah I.N., Allen P.D. (2009). Coordinated Movement of Cytoplasmic and Transmembrane Domains of RyR1 upon Gating. PLoS Biol..

[171] Pessah I.N., Cherednichenko G., Lein P.J. (2010). Minding the Calcium Store: Ryanodine Receptor Activation as a Convergent Mechanism of PCB Toxicity. Pharmacol. Ther..

[172] Howard A.S., Fitzpatrick R., Pessah I., Kostyniak P., Lein P.J. (2003). Polychlorinated Biphenyls Induce Caspase-Dependent Cell Death in Cultured Embryonic Rat Hippocampal but Not Cortical Neurons via Activation of the Ryanodine Receptor. Toxicol. Appl. Pharmacol..

[173] Murugesan P., Kanagaraj P., Yuvaraj S., Balasubramanian K., Aruldhas M.M., Arunakaran J. (2005). The Inhibitory Effects of Polychlorinated Biphenyl Aroclor 1254 on Leydig Cell LH Receptors, Steroidogenic Enzymes and Antioxidant Enzymes in Adult Rats. Reprod. Toxicol..

[174] Glauert H.P., Tharappel J.C., Lu Z., Stemm D., Banerjee S., Chan L.S., Lee E.Y., Lehmler H.-J., Robertson L.W., Spear B.T. (2008). Role of Oxidative Stress in the Promoting Activities of PCBs. Environ. Toxicol. Pharmacol..

[175] Lyng G.D., Seegal R.F. (2008). Polychlorinated Biphenyl-Induced Oxidative Stress in Organotypic Co-Cultures: Experimental Dopamine Depletion Prevents Reductions in GABA. NeuroToxicology.

[176] Duntas L.H. (2008). Environmental Factors and Autoimmune Thyroiditis. Nat. Rev. Endocrinol..

[177] Liu N., Rizzi N., Boveri L., Priori S.G. (2009). Ryanodine Receptor and Calsequestrin in Arrhythmogenesis: What We Have Learnt from Genetic Diseases and Transgenic Mice. J. Mol. Cell. Cardiol..

[178] Lee Y.-M., Jacobs D.R., Lee D.-H. (2018). Persistent Organic Pollutants and Type 2 Diabetes: A Critical Review of Review Articles. Front. Endocrinol..

[179] Buha Djordjevic A., Antonijevic E., Curcic M., Milovanovic V., Antonijevic B. (2020). Endocrine-Disrupting Mechanisms of Polychlorinated Biphenyls. Curr. Opin. Toxicol..

[180] Curtis S.W., Terrell M.L., Jacobson M.H., Cobb D.O., Jiang V.S., Neblett M.F., Gerkowicz S.A., Spencer J.B., Marder M.E., Barr D.B. (2019). Thyroid Hormone Levels Associate with Exposure to Polychlorinated Biphenyls and Polybrominated Biphenyls in Adults Exposed as Children. Environ. Health.

[181] Buck Louis G.M., Sundaram R., Schisterman E.F., Sweeney A.M., Lynch C.D., Gore-Langton R.E., Maisog J., Kim S., Chen Z., Barr D.B. (2013). Persistent Environmental Pollutants and Couple Fecundity: The LIFE Study. Environ. Health Perspect..

[182] He Q.-L., Zhang L., Liu S.-Z. (2021). Effects of Polychlorinated Biphenyls on Animal Reproductive Systems and Epigenetic Modifications. Bull. Environ. Contam. Toxicol..

[183] Klenov V., Flor S., Ganesan S., Adur M., Eti N., Iqbal K., Soares M.J., Ludewig G., Ross J.W., Robertson L.W. (2021). The Aryl Hydrocarbon Receptor Mediates Reproductive Toxicity of Polychlorinated Biphenyl Congener 126 in Rats. Toxicol. Appl. Pharmacol..

[184] Axelrad D.A., Goodman S., Woodruff T.J. (2009). PCB Body Burdens in US Women of Childbearing Age 2001–2002: An Evaluation of Alternate Summary Metrics of NHANES Data. Environ. Res..

[185] Meeker J.D., Maity A., Missmer S.A., Williams P.L., Mahalingaiah S., Ehrlich S., Berry K.F., Altshul L., Perry M.J., Cramer D.W. (2011). Serum Concentrations of Polychlorinated Biphenyls in Relation to in Vitro Fertilization Outcomes. Environ. Health Perspect..

[186] Huang Y., Yan M., Nie H., Wang W., Wang J. (2019). Persistent Halogenated Organic Pollutants in Follicular Fluid of Women Undergoing in vitro Fertilization from China: Occurrence, Congener Profiles, and Possible Sources. Environ. Pollut..

[187] Bloom M.S., Fujimoto V.Y., Storm R., Zhang L., Butts C.D., Sollohub D., Jansing R.L. (2017). Persistent Organic Pollutants (POPs) in Human Follicular Fluid and in vitro Fertilization Outcomes, a Pilot Study. Reprod. Toxicol..

[188] Patel S., Zhou C., Rattan S., Flaws J.A. (2015). Effects of Endocrine-Disrupting Chemicals on the Ovary. Biol. Reprod..

[189] Axmon A., Rylander L., Strömberg U., Hagmar L. (2004). Altered Menstrual Cycles in Women with a High Dietary Intake of Persistent Organochlorine Compounds. Chemosphere.

[190] Buck Louis G.M., Dmochowski J., Lynch C., Kostyniak P., McGuinness B.M., Vena J.E. (2009). Polychlorinated Biphenyl Serum Concentrations, Lifestyle and Time-to-Pregnancy. Hum. Reprod..

[191] Toft G., Thulstrup A.M., Jönsson B.A., Pedersen H.S., Ludwicki J.K., Zvezday V., Bonde J.P. (2010). Fetal Loss and Maternal Serum Levels of 2,2′,4,4′,5,5′-Hexachlorbiphenyl (CB-153) and 1,1-Dichloro-2,2-Bis(p-Chlorophenyl)Ethylene (p,p′-DDE) Exposure: A Cohort Study in Greenland and Two European Populations. Environ. Health.

[192] Grindler N.M., Allsworth J.E., Macones G.A., Kannan K., Roehl K.A., Cooper A.R. (2015). Persistent Organic Pollutants and Early Menopause in U.S. Women. PLoS ONE.

[193] Neblett M.F., Curtis S.W., Gerkowicz S.A., Spencer J.B., Terrell M.L., Jiang V.S., Marder M.E., Barr D.B., Marcus M., Smith A.K. (2020). Examining Reproductive Health Outcomes in Females Exposed to Polychlorinated Biphenyl and Polybrominated Biphenyl. Sci. Rep..

[194] Hauser R., Altshul L., Chen Z., Ryan L., Overstreet J., Schiff I., Christiani D.C. (2002). Environmental Organochlorines and Semen Quality: Results of a Pilot Study. Environ. Health Perspect..

[195] Dallinga J.W., Moonen E.J.C., Dumoulin J.C.M., Evers J.L.H., Geraedts J.P.M., Kleinjans J.C.S. (2002). Decreased Human Semen Quality and Organochlorine Compounds in Blood. Hum. Reprod..

[196] Mumford S.L., Kim S., Chen Z., Gore-Langton R.E., Boyd Barr D., Buck Louis G.M. (2015). Persistent Organic Pollutants and Semen Quality: The LIFE Study. Chemosphere.

[197] Sumner R.N., Tomlinson M., Craigon J., England G.C.W., Lea R.G. (2019). Independent and Combined Effects of Diethylhexyl Phthalate and Polychlorinated Biphenyl 153 on Sperm Quality in the Human and Dog. Sci. Rep..

[198] Jensen T.K. (2019). Endocrine Disrupters, Semen Quality and Anogenital Distance. Curr. Opin. Endocr. Metab. Res..

[199] Stukenborg J.-B., Mitchell R.T., Söder O. (2021). Endocrine Disruptors and the Male Reproductive System. Best Pract. Res. Clin. Endocrinol. Metab..

[200] Paoli D., Giannandrea F., Gallo M., Turci R., Cattaruzza M.S., Lombardo F., Lenzi A., Gandini L. (2015). Exposure to Polychlorinated Biphenyls and Hexachlorobenzene, Semen Quality and Testicular Cancer Risk. J. Endocrinol. Investig..

[201] Paul R., Moltó J., Ortuño N., Romero A., Bezos C., Aizpurua J., Gómez-Torres M.J. (2017). Relationship between Serum Dioxin-like Polychlorinated Biphenyls and Post-Testicular Maturation in Human Sperm. Reprod. Toxicol..

[202] Goncharov A., Rej R., Negoita S., Schymura M., Santiago-Rivera A., Morse G., Carpenter D.O., Akwesasne Task Force on the Environment (2009). Lower Serum Testosterone Associated with Elevated Polychlorinated Biphenyl Concentrations in Native American Men. Environ. Health Perspect..

[203] Paul R., Romero A., Moltó J., Ortuño N., Aizpurua J., Gómez-Torres M.J. (2021). Associations of Paternal Serum Dioxin-like Polychlorinated Biphenyl Concentrations with IVF Success: A Pilot Study. Environ. Res..

[204] Bush B., Bennett A.H., Snow J.T. (1986). Polychlorobiphenyl Congeners, p,p′-DDE, and Sperm Function in Humans. Arch. Environ. Contam. Toxicol..

[205] Bräuner E.V., Lim Y.-H., Koch T., Uldbjerg C.S., Gregersen L.S., Pedersen M.K., Frederiksen H., Petersen J.H., Coull B.A., Andersson A.-M. (2021). Endocrine Disrupting Chemicals and Risk of Testicular Cancer: A Systematic Review and Meta-Analysis. J. Clin. Endocrinol. Metab..

[206] Swan S.H., Colino S.A. (2021). Count Down—How Our Modern World Is Threatening Sperm Counts, Altering Male and Female Reproductive Development, and Imperiling the Future of the Human Race.

[207] Crinnion W.J. (2010). Toxic Effects of the Easily Avoidable Phthalates and Parabens. Altern. Med. Rev..

[208] Meeker J.D., Ehrlich S., Toth T.L., Wright D.L., Calafat A.M., Trisini A.T., Ye X., Hauser R. (2010). Semen Quality and Sperm DNA Damage in Relation to Urinary Bisphenol A among Men from an Infertility Clinic. Reprod. Toxicol..

[209] Meeker J.D., Yang T., Ye X., Calafat A.M., Hauser R. (2011). Urinary Concentrations of Parabens and Serum Hormone Levels, Semen Quality Parameters, and Sperm DNA Damage. Environ. Health Perspect..

[210] Li D.-K., Zhou Z., Miao M., He Y., Wang J., Ferber J., Herrinton L.J., Gao E., Yuan W. (2011). Urine Bisphenol-A (BPA) Level in Relation to Semen Quality. Fertil. Steril..

[211] Mori C., Nakamura N., Todaka E., Fujisaki T., Matsuno Y., Nakaoka H., Hanazato M. (2014). Correlation between Human Maternal–Fetal Placental Transfer and Molecular Weight of PCB and Dioxin Congeners/Isomers. Chemosphere.

[212] Mennigen J.A., Thompson L.M., Bell M., Tellez Santos M., Gore A.C. (2018). Transgenerational Effects of Polychlorinated Biphenyls: 1. Development and Physiology across 3 Generations of Rats. Environ. Health.

[213] Gore A.C., Thompson L.M., Bell M., Mennigen J.A. (2021). Transgenerational Effects of Polychlorinated Biphenyls: 2. Hypothalamic Gene Expression in Rats. Biol. Reprod..

[214] Gillette R., Son M.J., Ton L., Gore A.C., Crews D. (2018). Passing Experiences on to Future Generations: Endocrine Disruptors and Transgenerational Inheritance of Epimutations in Brain and Sperm. Epigenetics.

[215] Kezios K.L., Liu X., Cirillio P.M., Kalantzi O.I., Wang Y., Petreas M.X., Park J.-S., Bradwin G., Cohn B.A., Factor-Litvak P. (2012). Prenatal Polychlorinated Biphenyl Exposure Is Associated with Decreased Gestational Length but Not Birth Weight: Archived Samples from the Child Health and Development Studies Pregnancy Cohort. Environ. Health.

[216] Lignell S., Aune M., Darnerud P.O., Hanberg A., Larsson S.C., Glynn A. (2013). Prenatal Exposure to Polychlorinated Biphenyls (PCBs) and Polybrominated Diphenyl Ethers (PBDEs) May Influence Birth Weight among Infants in a Swedish Cohort with Background Exposure: A Cross-Sectional Study. Environ. Health.

[217] Papadopoulou E., Caspersen I.H., Kvalem H.E., Knutsen H.K., Duarte-Salles T., Alexander J., Meltzer H.M., Kogevinas M., Brantsæter A.L., Haugen M. (2013). Maternal Dietary Intake of Dioxins and Polychlorinated Biphenyls and Birth Size in the Norwegian Mother and Child Cohort Study (MoBa). Environ. Int..

[218] Caspersen I.H., Haugen M., Schjølberg S., Vejrup K., Knutsen H.K., Brantsæter A.L., Meltzer H.M., Alexander J., Magnus P., Kvalem H.E. (2016). Maternal Dietary Exposure to Dioxins and Polychlorinated Biphenyls (PCBs) Is Associated with Language Delay in 3year Old Norwegian Children. Environ. Int..

[219] Kristensen S.L., Ramlau-Hansen C.H., Ernst E., Olsen S.F., Bonde J.P., Vested A., Halldorsson T.I., Rantakokko P., Kiviranta H., Toft G. (2016). Prenatal Exposure to Persistent Organochlorine Pollutants and Female Reproductive Function in Young Adulthood. Environ. Int..

[220] Guo Y.L., Hsu P.-C., Hsu C.-C., Lambert G.H. (2000). Semen Quality after Prenatal Exposure to Polychlorinated Biphenyls and Dibenzofurans. Lancet.

[221] Lessard M., Herst P.M., Charest P.L., Navarro P., Joly-Beauparlant C., Droit A., Kimmins S., Trasler J., Benoit-Biancamano M.-O., MacFarlane A.J. (2019). Prenatal Exposure to Environmentally-Relevant Contaminants Perturbs Male Reproductive Parameters Across Multiple Generations That Are Partially Protected by Folic Acid Supplementation. Sci. Rep..

[222] Sheinberg R., Siegel E.L., Keidar R., Mandel D., Lubetzky R., Kohn E., Livneh A., Tovbin J., Betser M., Moskovich M. (2020). Associations between Intrauterine Exposure to Polychlorinated Biphenyls on Neonatal Ano-Genital Distance. Reprod. Toxicol..

[223] Raffetti E., Speziani F., Donato F., Leonardi L., Orizio G., Scarcella C., Apostoli P., Magoni M. (2017). Temporal Trends of Polychlorinated Biphenyls Serum Levels in Subjects Living in a Highly Polluted Area from 2003 to 2015: A Follow-up Study. Int. J. Hydrog. Energy Health.

[224] Spanò M., Toft G., Hagmar L., Eleuteri P., Rescia M., Rignell-Hydbom A., Tyrkiel E., Zvyezday V., Bonde J.P., INUENDO (2005). Exposure to PCB and p, P′-DDE in European and Inuit Populations: Impact on Human Sperm Chromatin Integrity. Hum. Reprod..

[225] Lettieri G., D’Agostino G., Mele E., Cardito C., Esposito R., Cimmino A., Giarra A., Trifuoggi M., Raimondo S., Notari T. (2020). Discovery of the Involvement in DNA Oxidative Damage of Human Sperm Nuclear Basic Proteins of Healthy Young Men Living in Polluted Areas. Int. J. Mol. Sci..

[226] Lettieri G., Marra F., Moriello C., Prisco M., Notari T., Trifuoggi M., Giarra A., Bosco L., Montano L., Piscopo M. (2020). Molecular Alterations in Spermatozoa of a Family Case Living in the Land of Fires—A First Look at Possible Transgenerational Effects of Pollutants. Int. J. Mol. Sci..

[227] Montano L., Donato F., Bianco P.M., Lettieri G., Guglielmino A., Motta O., Bonapace I.M., Piscopo M. (2021). Semen Quality as a Potential Susceptibility Indicator to SARS-CoV-2 Insults in Polluted Areas. Environ. Sci. Pollut. Res..

[228] Montano L., Donato F., Bianco P.M., Lettieri G., Guglielmino A., Motta O., Bonapace I.M., Piscopo M. (2021). Air Pollution and COVID-19: A Possible Dangerous Synergy for Male Fertility. Int. J. Environ. Res. Public Health.

[229] Raimondo S., Gentile M., Esposito G., Gentile T., Ferrara I., Crescenzo C., Palmieri M., Cuomo F., De Filippo S., Lettieri G. (2021). Could Kallikrein-Related Serine Peptidase 3 Be an Early Biomarker of Environmental Exposure in Young Women?. Int. J. Environ. Res. Public Health.

[230] Longo V., Forleo A., Radogna A.V., Siciliano P., Notari T., Pappalardo S., Piscopo M., Montano L., Capone S. (2022). A Novel Human Biomonitoring Study by Semiconductor Gas Sensors in Exposomics: Investigation of Health Risk in Contaminated Sites. Environ. Pollut..

[231] Montano L., Maugeri A., Volpe M.G., Micali S., Mirone V., Mantovani A., Navarra M., Piscopo M. (2022). Mediterranean Diet as a Shield against Male Infertility and Cancer Risk Induced by Environmental Pollutants: A Focus on Flavonoids. Int. J. Mol. Sci..

